# Adaptive Introgression across Species Boundaries in *Heliconius* Butterflies

**DOI:** 10.1371/journal.pgen.1002752

**Published:** 2012-06-21

**Authors:** Carolina Pardo-Diaz, Camilo Salazar, Simon W. Baxter, Claire Merot, Wilsea Figueiredo-Ready, Mathieu Joron, W. Owen McMillan, Chris D. Jiggins

**Affiliations:** 1 Department of Zoology, University of Cambridge, Cambridge, United Kingdom; 2 Smithsonian Tropical Research Institute, Panama, Panama; 3 CNRS UMR 7205, Muséum National d'Histoire Naturelle, CP50, Paris, France; 4 Universidade Federal do Pará, Instituto de Estudos Costeiros, Bragança, Brazil; Harvard University, United States of America

## Abstract

It is widely documented that hybridisation occurs between many closely related species, but the importance of introgression in adaptive evolution remains unclear, especially in animals. Here, we have examined the role of introgressive hybridisation in transferring adaptations between mimetic *Heliconius* butterflies, taking advantage of the recent identification of a gene regulating red wing patterns in this genus. By sequencing regions both linked and unlinked to the red colour locus, we found a region that displays an almost perfect genotype by phenotype association across four species, *H. melpomene*, *H. cydno*, *H. timareta*, and *H. heurippa*. This particular segment is located 70 kb downstream of the red colour specification gene *optix*, and coalescent analysis indicates repeated introgression of adaptive alleles from *H. melpomene* into the *H. cydno* species clade. Our analytical methods complement recent genome scale data for the same region and suggest adaptive introgression has a crucial role in generating adaptive wing colour diversity in this group of butterflies.

## Author Summary

Hybridisation occurs between many animal species, however its evolutionary relevance is still a matter of great debate. While some argue that hybridisation leads to maladaptive gene combinations, and therefore to an evolutionary dead end, others consider interspecific hybridisation as a process with great potential to fuel evolution. We examine this question by exploring the origins of red wing colouration, a trait under natural selection, in the adaptive radiation of closely related species of *Heliconius* butterflies. By sequencing genetic regions both linked and unlinked to the red wing pattern locus, we found experimental evidence supporting multiple hybridisation events that have mediated the acquisition of colour adaptations from *H. melpomene* to *H. timareta*. This introgression has allowed *H. timareta* to colonise new fitness peaks in the Müllerian mimicry landscape. In this way, our results support the idea that interspecific hybridisation in animals constitutes a source of genetic variation that promotes diversification.

## Introduction

Closely related species often hybridise through incomplete barriers to gene flow, but the evolutionary consequences of such genetic interchange remain a matter of debate [1], [2], [3], [4], [5], [6]. This is primarily because hybridisation is considered unlikely to introduce useful genetic variation [1], [4], [5], [7]. Alleles that cross species boundaries may be neutral in their effects [7] or, perhaps most commonly, natural selection will prevent the introgression of foreign genetic material into a genetic background that is already well adapted [8]. However, sometimes, introgression may be favoured if the region gained confers advantages to the recipient species [5]. Although such favourable gene combinations may be produced only rarely, they might still contribute important variation for adaptive change. Importantly, hybridisation is a potential source of novel alleles already tested by natural selection that would be unlikely to arise through mutation alone.

In organisms other than bacteria, evidence for adaptive introgression in nature is scarce [9], [10]. Nonetheless, several remarkable examples in plants have demonstrated adaptive introgression, for example in transferring herbivore resistance in *Helianthus* [5], flood tolerance in *Iris* [11] and the gene controlling rayed flowers in *Senecio vulgaris* [12]. In animals, examples include adaptive introgression of melanism from domestic dogs into North American wolves [13] and *warfarin* pesticide resistance in European house mice, gained from the Algerian mouse [14]. Nonetheless, these examples all represent a single instance of transfer of a trait, often in association with environments showing significant levels of human intervention. A more pervasive role for introgression in recent adaptive radiations has been postulated, for example in Darwin's finches and sailfins [15], [16], but convincing genetic evidence for introgression of specific adaptive traits is still missing in these systems.

*Heliconius* butterflies display a striking radiation in adaptive wing patterns, facilitated by Müllerian mimicry between distantly related species and coupled with divergence between closely related species [17]. These butterflies frequently hybridise across species boundaries [18], [19], and it has been hypothesised that introgression might play an important role in speciation and adaptive radiation. In particular two closely related species groups, *Heliconius melpomene* and *Heliconius cydno* are known to hybridise occasionally, and genetic evidence indicates a low level of ongoing gene flow [20], [21]. *H. melpomene* has radiated into almost 30 geographical colour pattern races across Central and South America [22], broadly falling into two main phenotypes, which we here refer to as the red-banded type (presence of a red band or patch in the forewing controlled by the *B* allele, regardless of hind wing phenotype) and the rays type (orange forewing basal patch and orange rays in the hind wing). The sister clade to *H. melpomene* includes the species *Heliconius cydno*, *H. pachinus*, *H. timareta* and *H. heurippa*, jointly referred to hereafter as the *H. cydno* clade [23]. The former two species are typically black with white or yellow elements [22], while the latter two species exhibit patterns similar to those of *H. melpomene* [23], [24]. We have previously suggested that the presence of red phenotype elements in these *H. cydno* affiliates, that is *H. heurippa* and *H. timareta*, could be the result of the acquisition of mimicry colour patterns via adaptive introgression from *H. melpomene* [3], [19], [24], and in the case of *H. heurippa* have provided DNA sequence evidence in support of this transfer [25]. However, these phenotypic patterns could also be explained if red variants were either ancestral, with multiple subsequent trait losses in the *H. cydno* clade, or if they had independent origins in both *H. melpomene* and the red *H. cydno* affiliates, specifically *H. timareta* and *H. heurippa* [26].

In *H. melpomene* the *HmB* locus controls variation in red colour patterns [27], [28], a trait under strong natural selection [29], [30]. Genomic analysis of this region has identified clear peaks of genetic divergence between adjacent races of *H. melpomene* associated with variation in red phenotypes [25], [28], [31]. In *H. melpomene*, the strongest divergence lies in a non-coding region in between a *kinesin* gene and the transcription factor *optix* [31]. The latter is the strongest candidate gene so far for the red locus [32], and its expression shows a perfect association with red wing colour elements in a wide range of geographical races of *H. melpomene* and its co-mimics *H. erato*, prefiguring in both species the forewing red band, the dennis orange patch and the hind wing rays [32].

Having such information provides an excellent opportunity to explicitly test the introgression hypothesis for red wing patterns across the broader *H. melpomene/H. cydno* species complex. Here, we specifically examine the phylogenetic history of divergent and convergent colour pattern races of *H. melpomene*, *H. cydno*, *H. timareta* and *H. heurippa* and ask how this history varies between loci linked and unlinked to colour pattern. The data allows us to understand the origins of adaptive colouration and ask whether similar wing patterns have multiple independent origins, or arose once within the complex and crossed species boundaries. Thus, we provide an explicit test of the hypothesis that hybridisation has repeatedly contributed to an adaptive radiation. This study was carried out alongside a genome-wide study of a subset of the taxa included here [33]. The analyses presented here on smaller gene regions, sequenced across a much larger set of taxa, permit a different set of analytical tools to be used to test for the extent and direction of introgression.

## Results

**Figure 1:**
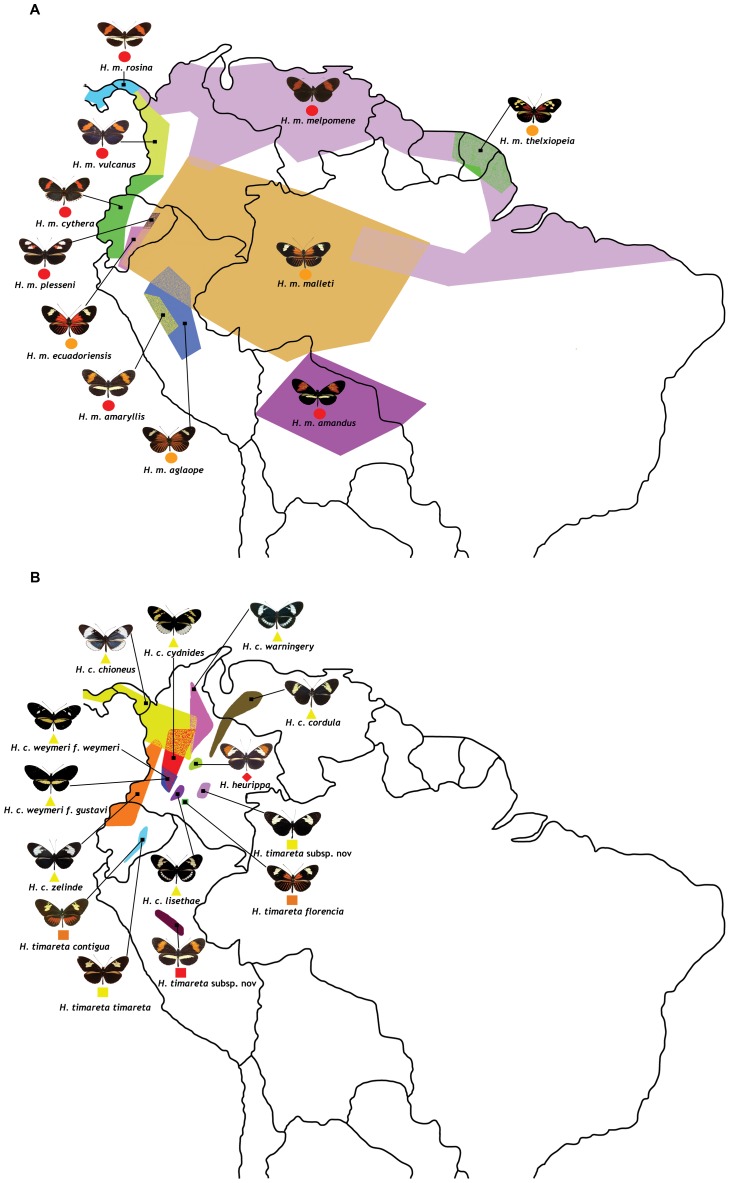
Geographic distribution and phenotype radiation of the species and races used in this study. (a) *H. melpomene* (circles) and (b) *H. cydno* (triangles), *H. timareta* (squares) and *H. heurippa* (diamonds). Taxa with a rayed phenotype are coloured orange, red-banded phenotypes (red band in the forewing) are red and, those taxa lacking a dorsal red wing phenotype are yellow. The distributions of sampled taxa are estimated from locality data compiled by Neil Rosser.

**Figure 2:**
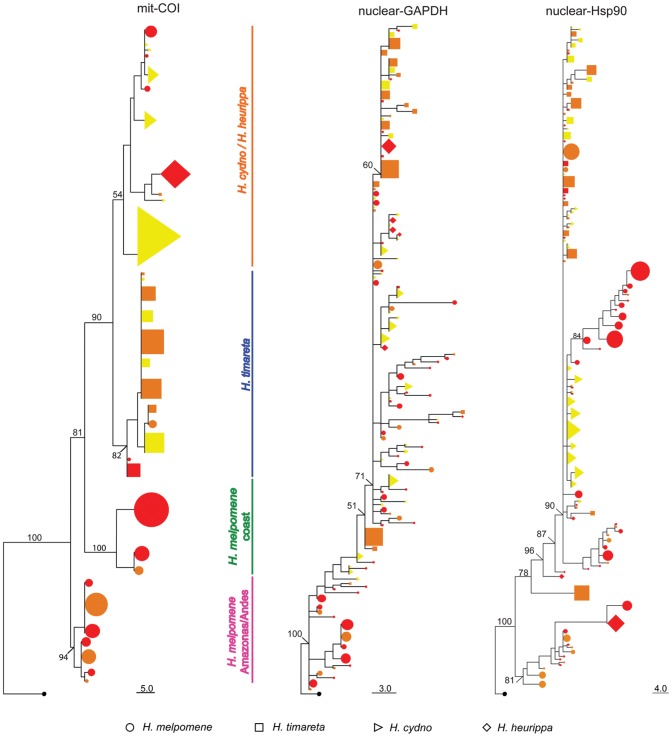
Phylogenetic clustering of unlinked markers. Neighbour-Joining tree of mitochondrial COI haplotypes and alleles at the nuclear loci *GAPDH* and *Hsp90*. Numbers above branches are the values for bootstrap support. Species and colour codes are as specified in Figure 1, with *H. melpomene* (circles) and *H. cydno* (triangles), *H. timareta* (squares) and *H. heurippa* (diamonds). *H. numata* (black) was used as the outgroup. Size of shapes represents haplotype frequency. Taxa with a rayed phenotype are coloured orange, red-banded phenotypes are red and those taxa lacking a dorsal red wing phenotype are yellow.

We analysed 221 haplotypes from nine loci (Table S1), sampled from 111 individuals in five species (Figure 1). Three loci (the mitochondrial fragment *COI* and nuclear *GAPDH* and *Hsp90*) were unlinked to colour pattern, whereas the remaining six loci were sampled across the genomic interval modulating red pattern variation, specifically where the highest genetic divergence peaks associated with variation in red phenotypes have been found in *H. melpomene* [31]. Analysis of molecular variance in the mitochondrial fragment *COI* showed population structure largely explained by species relationships (∼47%) and geography (∼30%) but less by colour phenotype (Table 1). Phylogenetic analysis supports three monophyletic clades: (i) *H. cydno-H. timareta*, (ii) *H. melpomene* from the Pacific and the Atlantic coast, and (iii) *H. melpomene* from the Amazonas and the Andes (Figure 2).

**Table 1:** Population structure as inferred by Analysis of Molecular Variance (AMOVA).

Gene	Type	Percentage of variation explained by		
		Colour pattern	Species	Geography
*COI*	Unlinked	12.06	46.85	29.3
		0.02542**	0.000001***	0.000001***
*Hsp90*		13.01	18.53	10.25
		0.00033***	0.000001***	0.01190**
*GAPDH*		1.23	14.45	14.63
		0.20734	0.000001***	0.00198***
*Kinesin*	Linked	1.91	8.48	1.61
		0.50860	0.04431*	0.38492
*Hm01012*		0.56	2.52	1.66
		0.21017	0.02151**	0.10251
*HmB_449k*		24.24	3.48	1.34
		0.000001***	0.12864	0.23413
*HmB_453k*		62.42	21.94	4.71
		0.000001***	0.02546**	0.47619
*Optix*		28.39	33.54	20.73
		0.000001***	0.000001***	0.00066**
*HmB_520k*		28.05	43.88	24.10
		0.00231**	0.000001***	0.00231**

Percentage of variance explained by phenotype, species and geography is presented for markers both unlinked (shadowed) and linked (non-shadowed) to red colour pattern.

p-value is indicated below each percentage of variation.

*: p<0.05,

**: p 0.05<0.000001,

***: p = 0.000001.

**Figure 3:**
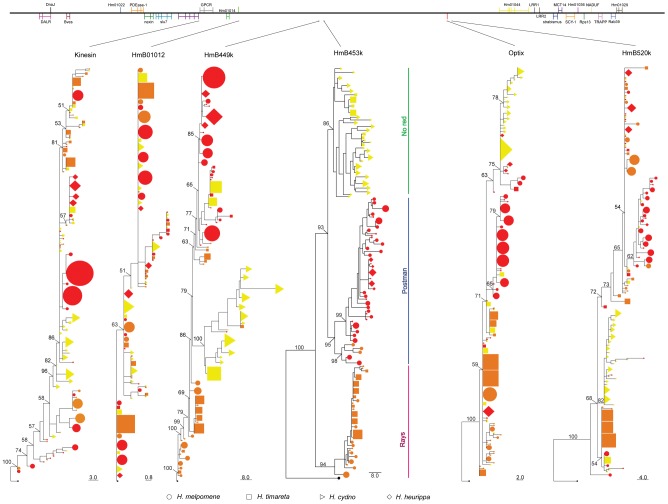
Phylogenetic clustering of the red colour linked markers. Top panel represents the order and gene content in the *HmB* red locus, with vertical lines representing exons. Lines drawn from top panel indicate the location of the marker in the *HmB* locus. All of the topologies were inferred by Neighbour-Joining clustering. Numbers above branches are the values for bootstrap support. Species and colour codes are as specified in Figure 1, with (a) *H. melpomene* (circles) and (b) *H. cydno* (triangles), *H. timareta* (squares) and *H. heurippa* (diamonds). *H. numata* (black) was used as outgroup. Size of shapes represents haplotype frequency. Taxa with a rayed phenotype are coloured orange, red-banded phenotypes are red and those taxa lacking a dorsal red wing phenotype are yellow.

In previous studies, nuclear markers showed varying degrees of clustering by species, with some loci showing mutual monophyly between the *H. melpomene* and *H. cydno* clade species, while others showed substantial allele sharing among species [20], [34]. Here, both unlinked nuclear markers (*GAPDH* and *Hsp90*) showed little population structure either by colour phenotype, species or geography (Table 1) with only about 15% of the variation explained by species and much less by colour pattern phenotype (Table 1). This result was corroborated by phylogenetic analysis (Figure 2), where similar alleles were spread broadly among species, wing pattern phenotypes, and across major biogeographic boundaries. Even among some loci within the red pattern interval, for instance *kinesin* and *Hm01012*, there was a poor correspondence with either species boundaries, geography or colour pattern, with each factor explaining less than 10% of the molecular variation at these markers (Table 1). Similarly, phylogenies of these two markers did not exhibit clear clustering by any of these categories (Figure 3).

Other markers across the red locus showed an increasing tendency to partition variation by colour pattern phenotype. Coding sequence of the transcription factor *optix* clustered most of the red-banded phenotypes of *H. melpomene* together, but *H. melpomene* individuals with rayed phenotypes were scattered across the genealogy. *Optix* also failed to show a clear phenotype association for *H. timareta* and *H. heurippa* (Figure 3). Nonetheless, colour pattern explained 28% of the variation within *optix* alleles (Table 1). A similar result was observed at *HmB449k* and *HmB520k*, which were 75 kb downstream and 2 kb upstream from *optix*, respectively, in the red interval (Table 1). Both loci grouped *H. melpomene* red-banded phenotypes into a monophyletic lineage (Figure 3), but failed to show a phenotype association in *H. heurippa*, *H. timareta* and rayed *H. melpomene*.

**Figure 4:**
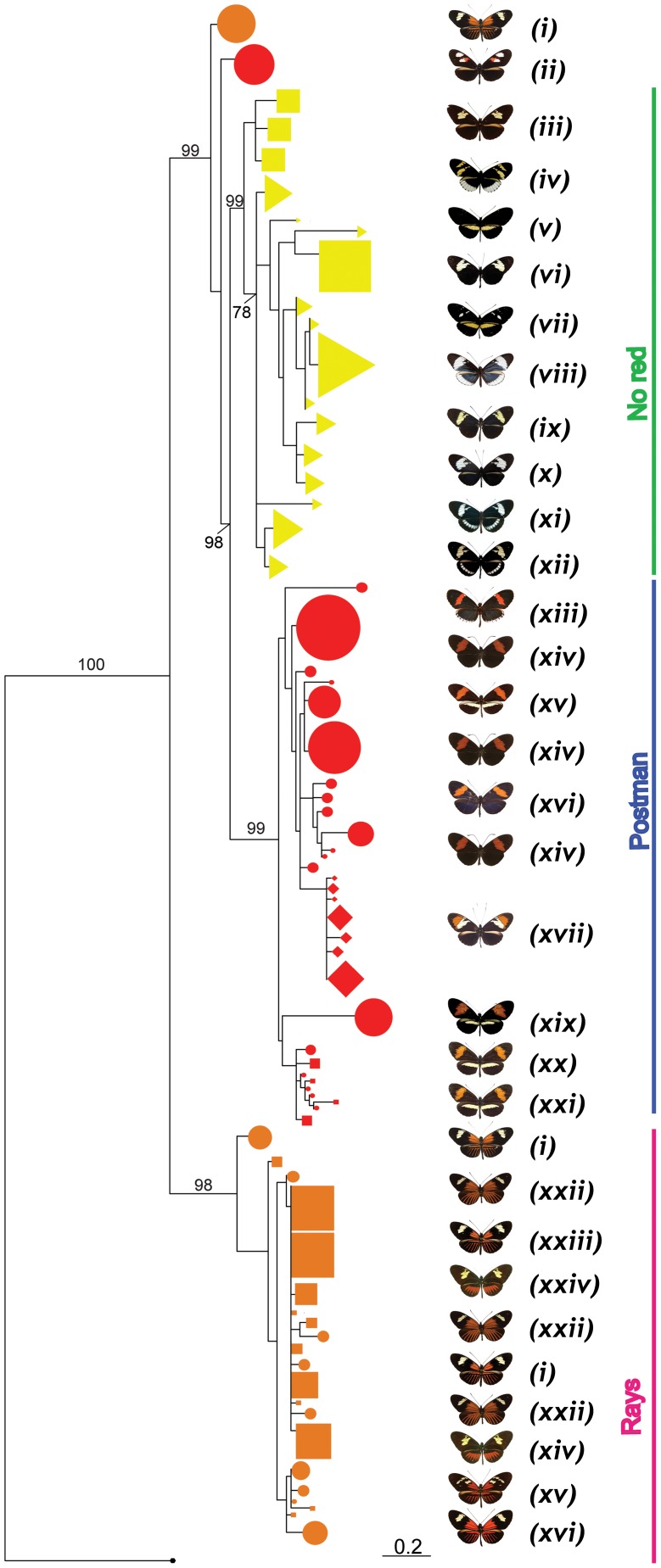
Phylogenetic clustering at HmB453k inferred by Maximum Likelihood. A phenotypic association regardless of species relationships is observed. Three major monophyletic clades (red, rays and no-red) are formed. Representative phenotypes of each clade are shown at the right (for full array of phenotypes and species see Figure 1). (i) *H. m. malleti*, (ii) *H. m. plesseni*, (iii) *H. t. timareta*, (iv) *H. c. cydnides*, (v) *H. c. weymeri* f. *gustavi*, (vi) *H. timareta* subsp. nov, (vii) *H. c. weymeri* f. *weymeri*, (viii) *H. c. chioneus*, (ix) *H. c. cordula*, (x) *H. c. zelinde*, (xi) *H. c. warningery*, (xii) *H. c. lisethae*, (xiii) *H. m. cythera*, (xiv) *H. m. melpomene*, (xv) *H. m. rosina*, (xvi) *H. m. vulcanus*, (xvii) *H. heurippa*, (xix) *H. m. amandus*, (xx) *H. m. amaryllis*, (xxi) *H. timareta* subsp. nov, (xxii) *H. m. aglaope*, (xxiii) *H. t. florencia*, (xxiv) *H. t. contigua*, (xxv) *H. m. thelxiopeia*, (xxvi) *H. m. ecuadoriensis*. Numbers above branches are the values for bootstrap support. *H. m. plesseni* and some of *H. m. malleti* (rayed phenotype) do not cluster within the three major phenotypic clades.

*HmB453k* was the striking exception to these patterns and showed strong population structure based on colour phenotype when analysed both by Neighbour-Joining and Maximum Likelihood (Figure 3, Figure 4). Over 60% of the segregating variation at this locus was explained by colour pattern phenotype (Table 1). Moreover, the allelic genealogy of this locus clearly defined three major clades, which largely corresponded to three major colour pattern phenotypes (Figure 4). The first clade contained red-banded type taxa (*H. melpomene*, *H. timareta* subsp. nov from Peru and *H. heurippa*), the second grouped rayed species (*H. melpomene*, *H. timareta florencia* and *H. timareta contigua*), and the third containing the species with no dorsal red wing colouration (*H. cydno*, *H. timareta* subsp. nov from Colombia and *H. timareta timareta*). Strikingly, individuals of the polymorphic population of *H. timareta* from eastern Ecuador were separated by phenotype, with rayed and non-rayed individuals sampled from the same locality falling into their respective phenotypic clades.

There were some exceptions to the complete clustering by phenotype in *HmB453k* (Figure 4). For example, the east Andean race, *H. m. plesseni* possesses white and red spots on the forewing and is typically considered a red-banded pattern. However, here all individuals from this race form a distinct monophyletic group on the *HmB453k* genealogy (Figure 4). This perhaps indicates that this phenotype shows an independent origin as compared to other red-banded patterns, consistent with its distinct white and red band phenotype. In addition, six haplotypes from the rayed race *H. m. malleti* did not cluster in the same clade as other rayed individuals, but similarly formed a separate monophyletic clade nested within the broader genealogy (Figure 4). This might also represent an independent origin of rayed phenotypes within *H. melpomene*, but is perhaps more likely a result of recombination between the *HmB453k* marker and nearby functional sites.

In order to address alternative explanations for the strong colour pattern signal within the *HmB453k* genealogy [26], we tested three alternative tree topologies for this fragment. The first alternate topology assumed that mtDNA topology correctly reflected the relationship among the three species; the second, a topology that considers independent phenotypic convergence in *H. melpomene*, *H. timareta* and *H. heurippa* and the third, a topology where *H. melpomene* constitutes a red polymorphic ancestral taxon and *H. cydno/H.timareta/H.heurippa* are derived with multiple losses of red patterns (Figure S1). According to the Shimodaira–Hasegawa (SH) test, the ML tree was better supported than any of the three alternative topologies (p<0.05 in all cases) [35]. These same three alternative tree topologies (Figure S1) were also tested against a ‘perfect’ ML *HmB453k* genealogy where the non-clustering alleles of *H. m. malleti* and *H. m. plesseni* were removed. In this case, again the SH test showed that the ML tree was better than any of the three alternatives (p<0.05 in all cases). Thus, we can rule out the alternative hypotheses proposed for pattern sharing across this group, namely multiple independent origins of red patterns, or ancestral red patterns subsequently lost multiple times [26].

To determine whether introgression is the cause of the shared DNA sequence variation observed among species, we applied the Isolation with Migration model in *H. melpomene*, *H. timareta* and *H. heurippa* using the program IM [36]. In order to obtain non-recombining blocks of sequence for this analysis, the taxa were separated into rayed and red-banded groups (see methods). In both datasets, IM estimated a population size of *H. timareta* smaller than that of *H. melpomene* (Table S3) and a time of divergence between these two species of ∼700,000 years. Maximum-likelihood estimates for introgression (2Nm), in general showed evidence of gene flow between species in the four markers analysed (Table 2). Models invoking gene flow in both directions were a significantly better fit than any model with no gene flow in any or in both directions (Table 3, models ABC0D, ABCD0, ABC00). We also found evidence for significant asymmetry in gene flow, as the model with unequal gene flow between species was significantly better than the model with similar gene flow in both directions (Table 3, model ABCDD).

**Table 2:** Rates of interspecies introgression between *H. melpomene*, *H. heurippa*, and *H. timareta*.

Gene		Red-banded		Rayed	
		*2N_1_m_1_*	*2N_2_m_2_*	*2N_1_m_1_*	*2N_2_m_2_*
*COI*	MLE	0.42	0.16	0.09	0.31
	Lower 90% HPD	0.11	0.01	0.01	0.11
	Upper 90% HPD	1.25	0.54	0.66	1.97
*GAPDH*	MLE	0.02	0.00	0.00	0.00
	Lower 90% HPD	0.01	0.00	0.00	0.00
	Upper 90% HPD	0.03	0.00	0.01	0.00
*Hsp90*	MLE	1.95	0.18	1.23	0.01
	Lower 90% HPD	1.56	0.12	0.78	0.00
	Upper 90% HPD	2.59	0.23	2.24	0.02
*HmB453k*	MLE	2.53	3.53	2.18	25.07
	Lower 90% HPD	0.81	1.80	0.79	1.64
	Upper 90% HPD	4.79	4.57	4.32	67.12

Migration rates were inferred using IM, with estimated values converted into effective units of population migration rate per generation assuming 10 generations per year. *m_1_* refers to migration from *H. timareta* to *H. melpomene* and *m_2_* refers to the migration from *H. melpomene* to *H. timareta*. The 2Nm values are the effective number of gene migrations received by a population per generation. Values of 2Nm greater than or equal to one can prevent populations from accumulating divergence [37].

Values of 2Nm in migrants per generation.

**Table 3:** Summary of likelihood ratio test statistics for the IMa analysis.

Dataset	Model	log (P)	2LLR	df	p value
Rays	ABC0D (m1 = 0)	−24.8052	68.2272	1	<0.001
	ABCD0 (m2 = 0)	−51.7291	122.0752	1	<0.001
	ABC00 (m1 = m2 = 0)	−57.9107	134.4383	2	<0.001
	ABCDD (m1 = m2>0)	−26.834	72.2849	1	<0.001
Postman	ABC0D (m1 = 0)	−11.6455	30.8555	1	<0.001
	ABCD0 (m2 = 0)	−154.1766	315.9178	1	<0.001
	ABC00 (m1 = m2 = 0)	−164.831	337.2264	2	<0.001
	ABCDD (m1 = m2>0)	−17.9431	43.4508	1	<0.001

Likelihood ratio statistics (2LLR), degrees of freedom (df) and significance level (P-value) when comparing the full likelihood model (ABCDE) to four different nested models. The results indicate significant support for bi-directional asymmetric gene flow in both data sets.

**Figure 5:**
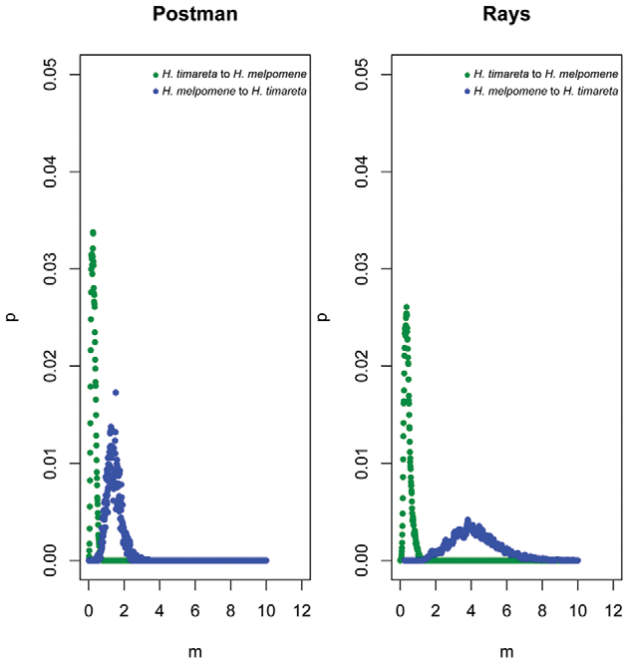
Directional introgression at *HmB453k*. Bayesian posterior probability distributions for the introgression rate (m) at *HmB453k* between *H. melpomene* and *H. timerata* in the rayed phenotypes, between *H. melpomene*, *H. timerata* and *H. heurippa* in the red-banded phenotype. Blue corresponds to introgression from *H. melpomene* to *H. timareta* whereas green corresponds to introgression in the other direction.

When gene flow parameters were estimated for individual genes, nuclear genes *Hsp90* and *GAPDH*, together with the mitochondrial fragment COI, showed evidence for ongoing gene flow between the study species (Table 2). However, the fragment *HmB453k* was the only marker consistently showing the strongest unidirectional introgression from *H. melpomene* to *H. timareta* in both phenotype datasets, thus suggesting that *HmB453k* alleles of *H. timareta* are derived from those of *H. melpomene* (Figure 5). Most notably in the rayed data set, this marker showed the highest magnitude of gene flow seen at any of the markers (Table 2). As the *HmB453k* fragment is located in the genomic region controlling the red wing phenotypes that is known to be under selection, one of the IM model assumptions is violated. Previous IM analysis on simulated scenarios with divergent selection in early stages of divergence have showed underestimated gene flow rates (2Nm) [37]. It could be argued therefore, that if selection is having an effect on our estimates we might be underestimating migration rates.

To further explore and confirm the signatures of introgression between these species we also used a linkage disequilibrium (LD) test for gene flow [38].

Briefly, the difference (*x* = D_SS_−D_SX_) between the magnitude of LD among all pairs of shared polymorphisms (D_SS;_ Disequilibrium Shared-Shared) and that among all pairs of sites for which one member is a shared polymorphism and the other is an exclusive polymorphism (D_SX;_ Disequilibrium Shared-Exclusive), is indicative of whether or not polymorphisms in the populations are the result of gene flow (positive *x* value) or retained ancestral polymorphism (negative *x* value) [38]. This because polymorphisms that are shared due to ancestral polymorphism are expected to be older on average, having more time to recombine and break down associations, than polymorphisms acquired via post-divergence gene flow [38].

We applied this test to the same phenotypic groups analysed with IM, and additionally, to pairs of *H. melpomene* and *H. timareta* populations found in sympatry. In general, the LD analysis showed values consistently positive across all comparisons and loci (Table 4), suggesting onging gene flow between *H. melpomene* and *H. timareta*. Notably, *HmB453k* was the only locus with significant gene flow in both the phenotypic and sympatric datasets, where *H. timareta* was always the recipient species (highest positive value) of *H. melpomene* alleles (p<0.001, Table 4). This analysis therefore provides strong confirmation of the IM results.

**Table 4:** Linkage disequilibrium test of historical gene flow.

	Phenotype	Locus	Observed	Simulated	p	Observed	Simulated	p
**Datasets by phenotype (as in IM)**			***H. timareta***			***H. melpomene***		
	Postman	*COI*	NA	0.258	NA	NA	0.45	NA
		*Hsp90*	0.122	0.014	<0.001	0.24	−0.008	<0.001
		*GAPDH*	0.405	0.019	<0.001	NA	0.069	NA
		*HmB453*	0.205	0.014	<0.001	0.056	−0.007	0.83
	Rays	*COI*	NA	1.299	NA	NA	1.488	NA
		*Hsp90*	0.166	0.275	0.78	0.225	0.079	0.21
		*GAPDH*	0.308	0.401	0.66	0.448	0.149	0.2
		*HmB453*	0.951	0.153	<0.001	0.256	0.04	0.04

	Location	Locus	Observed	Simulated	p	Observed	Simulated	p
**Datasets by sympatric populations**			***H. timareta*** ** subsp. nov**			***H. m. amaryllis***		
	Peru	*COI*	NA	NA	NA	NA	0.016	NA
		*Hsp90*	NA	0.2	NA	NA	0.022	NA
		*GAPDH*	0	0.018	0.64	0.126	0.122	1
		*HmB453k*	2.41	0.02	<0.001	0.035	0.0001	<0.001
			***H. t. timareta - H. t. contigua***			***H. m. malleti***		
	Ecuador	*COI*	NA	0.026	NA	NA	0.04	NA
		*Hsp90*	−0.114	0.003	1	−0.097	0.004	<0.001
		*GAPDH*	NA	0	NA	NA	0	NA
		*HmB453k*	0.478	0.004	<0.001	0.007	0.005	0.235
			***H. t. florencia***			***H. m. malleti***		
	South East- Colombia	*COI*	0	0.081	0.65	NA	NA	NA
		*Hsp90*	NA	0.014	NA	NA	0.018	NA
		*GAPDH*	0.36	0.002	<0.001	−0.283	0.002	1
		*HmB453k*	1.053	0.002	<0.001	−0.034	0.0001	0.98
			***H. heurippa***			***H. m. melpomene***		
	East Andes- Colombia	*COI*	NA	NA	NA	NA	NA	NA
		*Hsp90*	NA	0.0001	NA	NA	−0.005	NA
		*GAPDH*	−0.205	0.009	1	−0.255	0.011	1
		*HmB453k*	1	0.017	<0.001	0.55	0.015	<0.001

Observed and simulated values of *x = DSS-DSX* across four loci in *H. melpomene* and *H. timareta*. Positive values of *x* are indicative of gene flow and the recipient species displays the highest positive value. Simulated values of *x* were obtained with 30,000 simulations.

## Discussion

Adaptive novelty can arise *de novo* from mutations, from standing variation within populations or through gene flow among related populations or species, and the relative importance of these factors remains an open question in evolutionary biology. In *Heliconius* butterflies, the recent identification of the *optix* transcription factor as the locus of selection for red wing phenotypes offers the opportunity to address this question [32]. In a parallel study, we demonstrated that the distantly related *H. melpomene* and *H. erato* radiations use independently derived *optix* alleles to generate mimetic red patterns, implicating *de novo* mutations at the same locus [39]. Here, in contrast, we show that mimicry between more closely related species has involved multiple instances of allele sharing through adaptive introgression. Thus, the allelic variants that fuel adaptation do not necessarily need to be generated *de novo*, but can also be derived from introgression, accelerating the evolutionary process.

In this and previous studies, putatively neutral markers have shown that *H. melpomene* and the *H. cydno* clade are two distinct species assemblages that occasionally exchange genes [20], [21], [40]. Despite evidence for gene flow at neutral markers, *H. melpomene* and the *H. cydno* clade species often coexist in Central America and the Andes, and are ‘good’ species with distinct ecologies and strong barriers to gene flow, including both strong pre- and post-mating isolation [22], [41], [42], [43]. Here, we also found pervasive gene flow among *H. melpomene*, *H. timareta* and *H. heurippa*, similar to that previously observed in comparisons involving *H. melpomene* and *H. cydno* [21], [40]. The gene flow observed at markers unlinked to the wing pattern locus is bi-directional and not correlated with any obvious phenotypic trait. In contrast, the *HmB453k* marker, located within the red colour locus in a non-coding region downstream of *optix*, shows a striking association with wing phenotype and unidirectional introgression from *H. melpomene* to *H. timareta*.

The functional sites driving phenotypic variation within *Heliconius* are almost certainly *cis*-regulatory elements of *optix* and perhaps other adjacent protein coding regions, which act as a phenotypic switch for red pattern elements [32]. Notably, *optix* shows no amino acid substitutions between divergent colour pattern forms of the same species or between convergent forms of distantly related species [32]. To date, *HmB453k* shows the strongest association with wing pattern phenotype, much stronger than *kinesin*, which showed evidence for adaptive introgression of red phenotypes into *H. heurippa* [25], and even stronger than the *optix* coding region [32]. The strong signal we observe at *HmB453k* argues that it must be very close to the functional region(s) regulating colour pattern variation. Nonetheless, the fact that two races (*H. m. plesseni* and *H. m. malleti*) do not fall into the expected clades in this marker, might suggest that *HmB453k* does not itself contain functional sites. It is also likely that multiple functional sites across the genomic region control different aspects of the phenotype. Indeed, linkage disequilibrium analysis shows at least three sites in *optix* and *HmB453k* that consistently co-segregate (Figure S2), and in general there is substantial linkage disequilibrium across the *HmB* locus.

The lack of a strong association at the *kinesin* locus was surprising given the strong association seen at this locus in our previous study of *H. heurippa*, albeit with a much more taxonomically restricted sample [25]. However, we have considerably smaller sequence coverage of *kinesin* here, which might affect the signal we recovered from this gene. We believe that the previous study identified a genuine signal of introgression, but that the functional sites controlling the phenotype, which are likely to be regulatory in nature, are located in the non-coding sequence between *kinesin* and *optix*. Unpublished expression data indicate that there is evidence for functional involvement of both genes in wing pattern specification (Pardo-Diaz, unpublished data).

Previous criticism of the hypothesis of adaptive introgression in *Heliconius* and these species in particular, has focused on two alternative hypotheses, that either red variants were ancestral, with multiple subsequent trait losses, or that they have independent origins in these closely related lineages [26]. We have explicitly ruled out these alternatives, both by coalescent analysis using IM and LD analysis that indicate strong and significant evidence for directional gene flow, and by tree topology tests. In addition, it could also be hypothesised that natural selection might drive independent and convergent evolution of the sequence variants seen in the *HmB453k* region, if these were directly responsible for regulation of *optix* expression. Under this hypothesis however, one would expect multiple divergent haplotypes to be associated with this region in the surrounding sequence. Instead, we clearly observe a single haplotype at the centre of the associated region, with a decline in association with genetic distance, consistent with a single origin for each phenotype in this clade.

Alongside a parallel study involving a complete genomic sampling of the red colour region in a subset of the taxa used here [33], our data provide the first evidence for adaptive introgression driven by mimicry in *Heliconius*. The introgression previously documented in *H. heurippa* established a novel non-mimetic phenotype in eastern Colombia [24], [25]. In contrast, the additional cases of introgression documented here represent convergence due to mimicry selection, rather than establishment of an entirely novel pattern, albeit with a common genetic origin for the shared patterns. The direction of the asymmetrical gene flow is consistent with mimicry theory. First, *H. melpomene* is generally more locally abundant in the eastern Andes as compared to *H. timareta* (CJ, pers. obs.), so mimicry theory would predict that rare species should experience stronger selection to converge onto abundant models. Thus it seems likely that *H. timareta* adopted the local *H. melpomene* wing pattern, rather than vice-versa. Second, *H. cydno* and its co-mimics *H. sapho* and *H. eleuchia* are almost entirely restricted to the western side of the Andes [44]. One plausible scenario is therefore that the ancestors of *H. timareta* migrated along the eastern slope of the Andes and were faced with the absence of a white/yellow co-mimic. It seems likely that this imposed an additional selection pressure to mimic *H. melpomene* and *H. erato* instead. This eventually led to the establishment of *H. timareta* as a replacement of *H. cydno* distributed along the eastern slopes of the Andes in sympatry with *H. melpomene*.

The data provide evidence for multiple independent introgression events. *H. t. florencia* shares a rayed pattern with its co-mimic, *H. m. malleti*, in south-eastern Colombia [45], while the very different phenotype of the red-banded race *H. t. ssp. nov.* is mimetic with *H. m. amaryllis* in the Tarapoto region of Peru. A likely additional case is represented by the polymorphic population of *H. timareta* in Ecuador. Although the rayed phenotype in this population may share a common origin with that of *H. t. florencia* in Colombia, their distribution is disjunct, separated by the red banded *H. tristero* found in Mocoa, Colombia. Thus, the acquisition of red patterns by *H. timareta* has been driven by natural selection for mimicry, and has occurred multiple times (at least once for each red colour element) in the last 700,000 years.

The introgression of regions controlling red wing colouration from *H. melpomene* to the *H. cydno* clade has facilitated mimicry and has also played a role in speciation. In *H. heurippa* the red/yellow hybrid pattern is used as mating cue, which contributes to reproductive isolation from its closest relatives [24], [25], [46]. Although barriers to gene flow within *H. timareta* have not been investigated, it is possible that similar isolation might be found between red-banded and rayed races of this species, such that these might represent incipient species generated through hybridisation.

In this and previous work we are beginning to piece together a more complete picture of the history of this complex adaptive radiation. It seems likely that the red-banded pattern in *H. erato* spread and diversified early in the history of the radiation, followed by emergence of the *H. erato* rayed pattern that spread across Amazonia interrupting the geographical continuity of the ancestral red-banded phenotype [39]. In the *H. melpomene* lineage there was a speciation event in which *H. cydno* colonised the yellow/white phenotypic niche to mimic the *H. eleuchia* and *H. sapho* clade, and *H. melpomene* diversified to mimic the phylogenetically distant *H. erato* [39]. Reproductive isolation between the species is partly due to colour pattern mate choice, which arose between closely related taxa such as *H. melpomene* and *H. cydno*. Then divergence of the *H. timareta/heurippa* lineage from the rest of *H. cydno*, around 700,000 years ago, arose as a result of adaptive introgression of wing patterning alleles from *H. melpomene* in the eastern Andes.

In summary, we provide evidence that contributes to resolving the longstanding debate over the evolutionary importance of hybridisation in animals. Our data allow statistical tests for the incidence of introgression based on both coalescent patterns and linkage disequilibrium, with consistent results, and indicate the direction of introgression. The results imply that interspecific hybridisation facilitates adaptability and diversification, not only when the selection pressure is human-mediated, but also allows the colonisation of either empty or under-utilised fitness peaks in animal adaptive radiations. In other adaptive radiations such as Darwin's finches [15], *Daphnia* waterfleas [47] and African cichlids [48], rapid diversification may similarly be mediated by introgression [1]. The evolutionary impact of such transfers might be higher if the traits interchanged are also involved in reproductive isolation, thus contributing to speciation.

## Materials and Methods

### Sampling, amplification, and DNA sequencing

Our sample set consisted of 111 individuals from 4 different species, namely *H. melpomene*, *H. cydno*, *H. timareta* and *H. heurippa* (Table S1). In total 14 races of *H. melpomene*, 8 races of *H. cydno*, 5 races of *H. timareta* and one of *H. heurippa* were sampled covering most of the geographic distribution of each species from Central to South America (Figure 1). *H. numata* was included as outgroup. DNA was extracted using the QIAGEN DNeasy 96 Blood & Tissue Kit. One mitochondrial and eight nuclear fragments (Table S2) were amplified with QIAGEN Taq DNA Polymerase, purified using ExoSAP and sequenced with ABI Big Dye Terminator. Two of the nuclear markers are unlinked to the *HmB* red locus whereas the remaining six are all located across the region (Table S2). From these colour-linked fragments, *optix* and *kinesin* have previously been implicated in red wing pattern determination. The remaining four were identified as regions under divergent selection with high levels of population differentiation associated with red colouration [31].

Sequences were aligned and cleaned using Codon Code Aligner. Haplotype inference for heterozygous calls was conducted with the PHASE algorithm implemented in DNAsp v5.10.01 [49], with 5000 iterations and allowing for recombination. Inferred haplotypes with a confidence higher than 95% were accepted. In the case of the fragments *HmB449k* and *HmB453k* cloning was necessary due to the presence of considerable indel variation. PCR products of these two markers were ligated into the pGEM-T easy vector and five to ten clones per individual were selected and sequenced. Sequences were deposited in GenBank under accession numbers JX003980–JX005837.

### Phylogenetic reconstruction

For each fragment, phased haplotypes were used to construct phylogenetic trees using the Neighbour-Joining method under the P model of uncorrected distance in PAUP* 4.0b10. Node support in the resulting trees was estimated by 1000 bootstrap replicates using a heuristic search. To confirm the phylogenetic groupings obtained by Neighbour-Joining for *HmB453K*, a maximum likelihood phylogeny was also constructed with PhyML [50], using the GTR+I+G substitution model selected by Modeltest [51] and with branch support values obtained by 1000 bootstrap replicates. The stability of the inferred phylogeny for *HmB453k* was examined using the Shimodaira-Hasegawa test (SH test) [35] in PAUP* 4.0b10. For all phylogenetic inferences trees were rooted with *H. numata* as outgroup.

### Genetic structure analysis

Analysis of molecular variance (AMOVA) with 1000 permutations, implemented in ARLEQUIN v.3.5 [52], was used to assess population structure by species, geography and phenotype. For species, four groups were set, corresponding to *H. melpomene*, *H. cydno*, *H. timareta* and *H. heurippa*. In the geography analysis, haplotypes were grouped into six geographic regions: (i) the Guiana shield, (ii) Amazon, (iii) Pacific, (iv) East Andes foothills, (v) Cauca Valley and (vi) Magdalena Valley. These geographic clustering matches the biogeographic provinces (i) Humid Guyana, (ii) Napo+Imeri, (iii) Choco+Wester Ecuador+Arid Ecuador, (iv) North Andean Paramo, (v) Cauca and (vi) Magdalena defined by Morrone [53]. When compared by phenotype, haplotypes were grouped in three groups: the red-banded type [presence of red forewing band], the rayed type [presence of orange rays in the hind wing] and the non-red type [absence of any dorsal red element on the wings]. The outgroup *H. numata* was excluded from these analyses.

### Analysis of gene flow with the Isolation with Migration (IM) model

In order to estimate the role and direction of historical gene flow between *H. melpomene* and *H. timareta* (*H. heurippa* was included in *H. timareta* for the purposes of this analysis), we used the Isolation-Migration (IM) Bayesian model [36]. IM uses Markov chain Monte Carlo (MCMC) sampling to obtain maximum-likelihood estimates of six parameters: current population sizes, ancestral population size, rates of migration between two populations (*m*_1_ and *m*_2_) and the timing of divergence (*t*). IM assumes both free recombination between loci and no recombination within them, therefore the software SITES [54] was used to select genetic blocks with no recombination within each locus. To fulfill the assumption of free recombination between loci, only the unlinked colour loci and one of the fragments linked to red colouration [*HmB453k*] were selected for this analysis. We ran IM on two modified datasets for each species pair: (i) *H. melpomene* rayed type*-H. timareta* and (ii) *H. melpomene* red-banded -[*H. heurippa* and *H. timareta*]. These groups constituted the maximal units in which we could get enough data without recombination, with the rayed dataset being a block of 379 bp and the postman dataset one of 313 bp. Unfortunately, pairwise comparisons involving all the species' alleles were not possible nor were comparisons involving species in parapatry because such groupings contained small non-recombining blocks that lacked enough informative sites. However, since our main interest was to determine the direction and magnitude of introgression (*m*) within phenotypes, these datasets are sufficient for addressing this question. For all datasets, after searching for the parameter range using preliminary runs, 30 million steps were sampled from the primary chain after a 300,000 burn-in period under the HKY model with 10 chains per set. Mixing properties of the MCMC were assessed by visual inspection of the parameter trend plots and by examining that the effective sample size (ESS) was higher than 50, as recommended [36], [37].

To get biologically meaningful units of gene flow, the maximum likelihood estimates and 90% highest posterior density (HPD) interval for the migration rates (m) were converted into the effective number of gene migrations received by a population per generation (2Nm, in Table 2). For this conversion, we used a generation time of 35 days and a mutation rate per gene calculated with the calibration time proposed by Wahlberg et al. for Nymphalidae [55] coupled with the divergence between the melpomene/cydno clade per locus estimated with the software SITES. Our estimates of mutation rate per locus per year were: 6.2×10^−6^ for COI, 7.1×10^−8^ for GAPDH, 1.2×10^−6^ for Hsp90 and 3.4×10^−5^ for HmB453k.

We finally compared the model including all six parameters to simpler demographic models in order to statistically test the hypothesis of zero or equal gene flow between populations (m1 = 0, m2 = 0, m1 = m2 = 0, m1 = m2>0). These analyses were conducted using IMa [36] by running the initial M-mode output with identical settings in the L-mode and sampling 5×10^5^ genealogies.

### Analysis of gene flow based on linkage disequilibrium (LD) patterns

We further tested the presence, significance and direction of gene flow per locus between *H. melpomene* and *H. timareta* using a method based on linkage disequilibrium (LD) developed by Machado *et al* in 2002 [38]. In this test, a positive difference between the LD among all pairs of shared polymorphisms (D_SS_) and the LD among all pairs of sites for which one member is a shared polymorphism and the other is an exclusive polymorphism (D_SX_), is indicative of gene flow. The magnitude of the difference directly measures the direction of the introgression, with the species with the highest positive value being the recipient [38]. The same phenotypic datasets used in the IM analysis and also groups of species in complete sympatry, were subjected to independent runs, each of them with 30000 simulations. The input files were prepared with the program SITES [56] calculating D' as a measure of linkage disequilibrium (as suggested by Machado et. al [38]) and analysing linkage disequilibrium among shared polymorphism between groups (by choosing the -s and -p options in the LD string).

### Analysis of linkage disequilibrium on the HmB locus

Linkage disequilibrium across the *HmB* region was calculated for all populations using the software MIDAS [57] only considering sites with allele frequency higher than 5%, and visualised with the R package LDheatmap [58].

## Supporting Information

Figure S1 Schematic representation of the contrasting topologies used in the SH test. (a) Species tree inferred from mtDNA and (b) ‘independent phenotypic convergence’ tree where red-banded (red cross) and rays (orange cross) evolved independently in *H. melpomene*, *H. timareta* and *H. heurippa* (c) ‘Ancestral polymorphism’ tree, where *H. melpomene* acts as an ancestral red polymorphic taxa and *H. cydno*, *H. heurippa* and *H. timareta* are derived taxa where multiple retentions and losses (red crosses) of red traits have occurred.
(TIF)

Figure S2 Pairwise linkage disequilibrium.
(TIF)

Table S1 List of species, races, and specimens used.
(DOC)

Table S2 Primers and PCR information.
(DOC)

Table S3 Population size parameter estimated by IM.
(DOC)
