# Supplementary figures and images for: Adaptive Introgression across Species Boundaries in Heliconius Butterflies
Source: PLoS Genet. 2012 Jun 21;8(6):e1002752. doi: 10.1371/journal.pgen.1002752 (PMC3380824; doi:10.1371/journal.pgen.1002752)

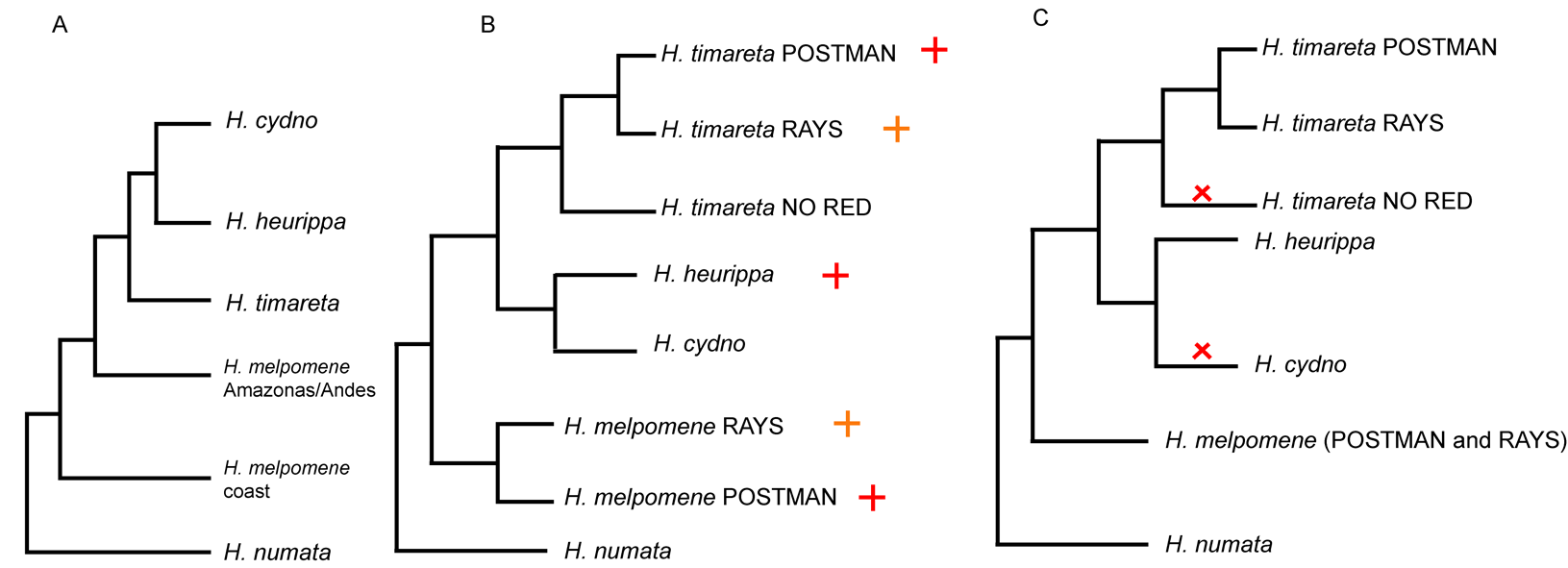

Supplement: Figure S1 — Schematic representation of the contrasting topologies used in the SH test. (a) Species tree inferred from mtDNA and (b) ‘independent phenotypic convergence’ tree where red-banded (red cross) and rays (orange cross) evolved independently in H. melpomene, H. timareta and H. heurippa (c) ‘Ancestral polymorphism’ tree, where H. melpomene acts as an ancestral red polymorphic taxa and H. cydno, H. heurippa and H. timareta are derived taxa where multiple retentions and losses (red crosses) of red traits have occurred. (TIF) [file pgen.1002752.s001.tif]

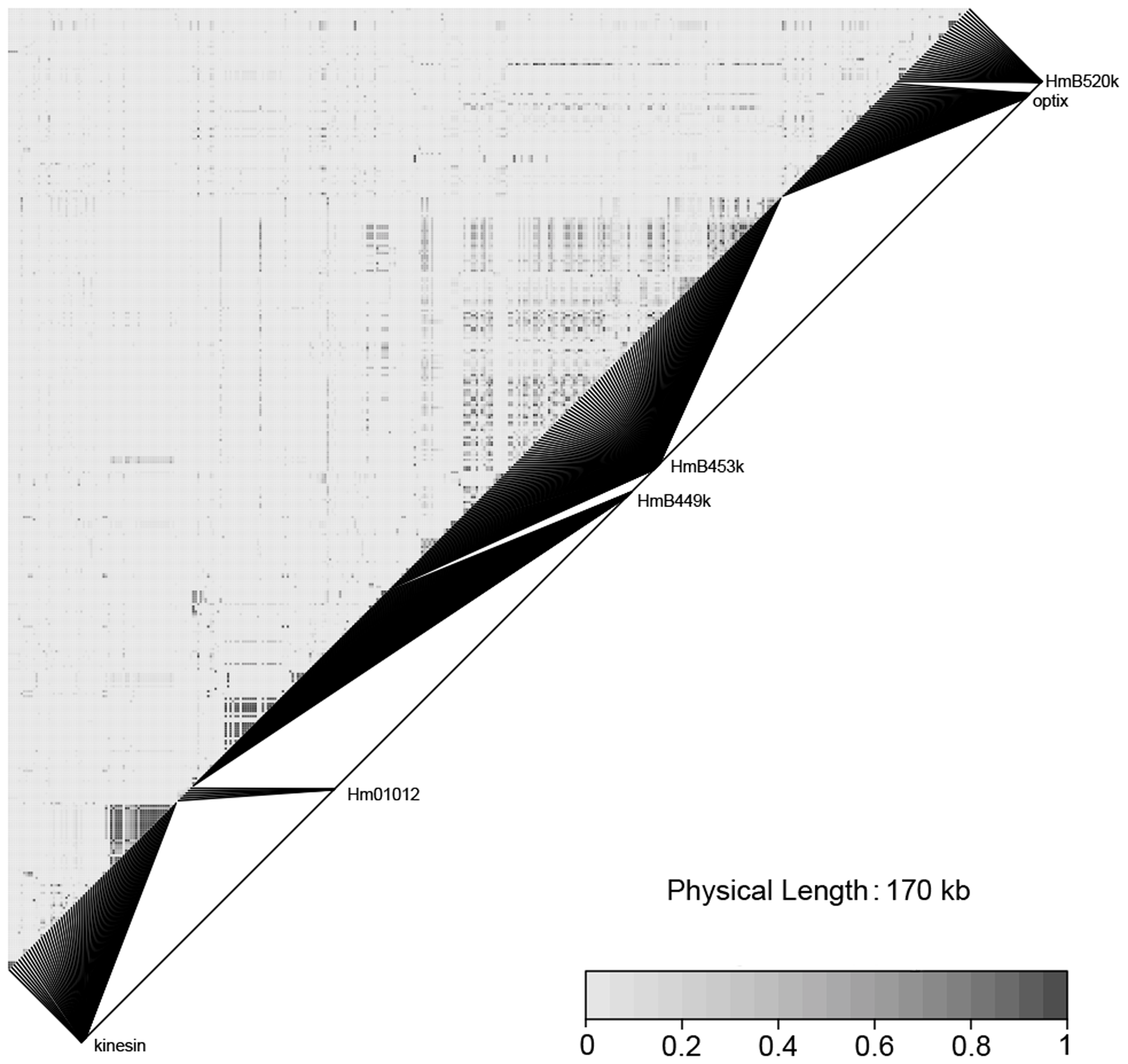

Supplement: Figure S2 — Pairwise linkage disequilibrium. (TIF) [file pgen.1002752.s002.tif]
