# Supplementary material for: Adaptive Introgression across Species Boundaries in Heliconius Butterflies
Source: PLoS Genet. 2012 Jun 21;8(6):e1002752. doi: 10.1371/journal.pgen.1002752 (PMC3380824; doi:10.1371/journal.pgen.1002752)
Supplement: Table S1 — List of species, races, and specimens used. (DOC) [file pgen.1002752.s003.doc]

**Table S1. Specimen and collection information**

| **Species** | **Race** | **Specimen number** | **Country** | **Locality** | **Coordinates** | **Geographic region (as in AMOVA)** |  |
| --- | --- | --- | --- | --- | --- | --- | --- |
| *Heliconius numata* |  | 2387 | Colombia | Quebrada Carbona, Curaño (Caqueta) | 1° N 75° W |  |  |
| *Heliconius heurippa* |  | 2205 | Colombia | Buenavista (Meta) | 4° N 73° W | East Andes foothills |  |
|  |  | 2417 | Colombia | Buenavista (Meta) | 4° N 73° W |  |  |
|  |  | 2150 | Colombia | Cubarral-Vereda el Vergel Alto (Meta) | 3° N 73° W |  |  |
|  |  | ch7 | Colombia | Buenavista (Meta) | 4° N 73° W |  |  |
|  |  | 2207 | Colombia | Buenavista (Meta) | 4° N 73° W |  |  |
|  |  | ch10 | Colombia | Buenavista (Meta) | 4° N 73° W |  |  |
|  |  | ch12 | Colombia | Buenavista (Meta) | 4° N 73° W |  |  |
|  |  | ch3 | Colombia | Buenavista (Meta) | 4° N 73° W |  |  |
|  |  | ch8 | Colombia | Buenavista (Meta) | 4° N 73° W |  |  |
| *Heliconius cydno* | *cordula* | 2822 | Venezuela | San Cristobal (UNET-Paramillo) | 7° N 72° W | East Andes foothills |  |
|  |  | 2182 | Venezuela | San Cristobal (UNET-Paramillo) | 7° N 72° W |  |  |
|  |  | 2253 | Venezuela | San Cristobal (UNET-Paramillo) | 7° N 72° W |  |  |
|  |  | 2823 | Venezuela | San Cristobal (UNET-Paramillo) | 7° N 72° W |  |  |
|  | *chinoeus* | 2440 | Panama | Pipeline road 5.2km | 9° N 79° W | Pacific |  |
|  |  | 8265 | Panama | Cerro Campana | 8.6° N 79.9° W |  |  |
|  |  | 2424 | Panama | Pipeline road 5.2km | 9° N 79° W |  |  |
|  | *cydnides* | 2017 | Colombia | Marsella-el nudo (Risaralda) | 4° N 75° W | Cauca Valley |  |
|  |  | 2020 | Colombia | Marsella-el nudo (Risaralda) | 4° N 75° W |  |  |
|  |  | 2023 | Colombia | Marsella-el nudo (Risaralda) | 4° N 75° W |  |  |
|  | *zelinde* | 2242 | Colombia | Ladrilleros (Valle del Cauca) | 3° N 77° W | Pacific |  |
|  |  | 2262 | Colombia | Ladrilleros (Valle del Cauca) | 3° N 77° W |  |  |
|  |  | 2264 | Colombia | Ladrilleros (Valle del Cauca) | 3° N 77° W |  |  |
|  | *weymeri f. gustavi* | 2478 | Colombia | Helechaux (Cauca) | 2° N 76° W | Cauca Valley |  |
|  |  | 2484 | Colombia | Helechaux (Cauca) | 2° N 76° W |  |  |
|  |  | 2527 | Colombia | Helechaux (Cauca) | 2° N 76° W |  |  |
|  | *weymeri f. weymeri* | 1906 | Colombia | Helechaux (Cauca) | 2° N 76° W | Cauca Valley |  |
|  |  | 2475 | Colombia | Helechaux (Cauca) | 2° N 76° W |  |  |
|  |  | 1911 | Colombia | Helechaux (Cauca) | 2° N 76° W |  |  |
|  | *lisethae* | 2370 | Colombia | San Agustin, Parque Arqueológico (Huila) | 1° N 76° W | Magdalena Valley |  |
|  |  | 2375 | Colombia | San Agustin, Parque Arqueológico (Huila) | 1° N 76° W |  |  |
|  |  | 2377 | Colombia | San Agustin, Parque Arqueológico (Huila) | 1° N 76° W |  |  |
|  | *weningery* | 2964 | Colombia | Tolota (Santander) | 6° N 73° W | Magdalena Valley |  |
|  |  | 2967 | Colombia | Tolota (Santander) | 6° N 73° W |  |  |
|  |  | 2970 | Colombia | Tolota (Santander) | 6° N 73° W |  |  |
| *Heliconius timareta* | subsp. nov | 2234 | Colombia | Las Morras Puerto Amor (Caqueta) | 2° N 74° W | East Andes foothills |  |
|  |  | 2434 | Colombia | Las Morras Puerto Amor (Caqueta) | 2° N 74° W |  |  |
|  |  | 2409 | Colombia | Las Morras Puerto Amor (Caqueta) | 2° N 74° W |  |  |
|  | *contigua* | 9174 | Ecuador | El Topo, Puyo Road | 1.4° S 78° W | East Andes foothills |  |
|  |  | 9179 | Ecuador | El Topo, Puyo Road | 1.4° S 78° W |  |  |
|  |  | 9181 | Ecuador | El Topo, Puyo Road | 1.4° S 78° W |  |  |
|  |  | 9187 | Ecuador | El Topo, Puyo Road | 1.4° S 78° W |  |  |
|  |  | 9224 | Ecuador | Rio Anso, near Mera | 1° S 78° W |  |  |
|  |  | 9225 | Ecuador | Rio Anso, near Mera | 1° S 78° W |  |  |
|  |  | 9170 | Ecuador | El Topo, Puyo Road | 1.4° S 78° W |  |  |
|  |  | 9171 | Ecuador | El Topo, Puyo Road | 1.4° S 78° W |  |  |
|  |  | 9173 | Ecuador | El Topo, Puyo Road | 1.4° S 78° W |  |  |
|  |  | 9177 | Ecuador | El Topo, Puyo Road | 1.4° S 78° W |  |  |
|  |  | 9180 | Ecuador | El Topo, Puyo Road | 1.4° S 78° W |  |  |
|  |  | 9185 | Ecuador | El Topo, Puyo Road | 1.4° S 78° W |  |  |
|  |  | 9223 | Ecuador | Rio Anso, near Mera | 1° S 78° W |  |  |
|  |  | 9172 | Ecuador | El Topo, Puyo Road | 1.4° S 78° W |  |  |
|  |  | 9164 | Ecuador | El Topo, Puyo Road | 1.4° S 78° W |  |  |
|  |  | 9176 | Ecuador | El Topo, Puyo Road | 1.4° S 78° W |  |  |
|  | *timareta* | 9169 | Ecuador | El Topo, Puyo Road | 1.4° S 78° W | East Andes foothills |  |
|  |  | 9182 | Ecuador | El Topo, Puyo Road | 1.4° S 78° W |  |  |
|  |  | 9183 | Ecuador | El Topo, Puyo Road | 1.4° S 78° W |  |  |
|  |  | 9184 | Ecuador | El Topo, Puyo Road | 1.4° S 78° W |  |  |
|  |  | 9226 | Ecuador | Rio Anso, near Mera | 1° S 78° W |  |  |
|  |  | 9227 | Ecuador | Rio Anso, near Mera | 1° S 78° W |  |  |
|  | *florencia* | 2402 | Colombia | Quebrada Doraditas, Suaza (Caqueta) | 1° N 75° W | East Andes foothills |  |
|  |  | 2406 | Colombia | Quebrada Doraditas, Suaza (Caqueta) | 1° N 75° W |  |  |
|  |  | 2410 | Colombia | Quebrada Doraditas, Suaza (Caqueta) | 1° N 75° W |  |  |
|  | subsp. nov | 8620 | Peru | La Antena, Km 15-24, Via Tarapoto-Yurinaguas Km17 | 6.4° S 76.3° W | East Andes foothills |  |
|  |  | 8629 | Peru | La Antena, Km 15-24, Via Tarapoto-Yurinaguas Km18 | 6.4° S 76.3° W |  |  |
|  |  | 8622 | Peru | La Antena, Km 15-24, Via Tarapoto-Yurinaguas Km19 | 6.4° S 76.3° W |  |  |
| *Heliconius melpomene* | *melpomene* | cm8 | Colombia | Morcote (Casanare) | 5° N 72° W | East Andes foothills |  |
|  |  | cm2 | Colombia | Morcote (Casanare) | 5° N 72° W |  |  |
|  |  | cm12 | Colombia | Morcote (Casanare) | 5° N 72° W |  |  |
|  |  | cm11 | Colombia | Morcote (Casanare) | 5° N 72° W |  |  |
|  |  | 1742 | Colombia | Chirajara (Cundinamarca) | 4° N 73° W |  |  |
|  |  | 2251 | Venezuela | San Cristobal (UNET-Paramillo) | 7° N 72° W |  |  |
|  |  | 14631 | Panama | Cana, around runway area | 7.7° N 77.7° W | Pacific |  |
|  |  | 14744 | Panama | Cana, around runway area | 7.7° N 77.7° W |  |  |
|  |  | 114677 | Panama | Cana, around runway area | 7.7° N 77.7° W |  |  |
|  |  | 8221 | French Guiana | Savane du Galion | 4.7° N 52.4° W | Guiana shield |  |
|  |  | 8239 | French Guiana | Pointe Macouria | 4.9° N 52.3° W |  |  |
|  |  | 8229 | French Guiana | Savane du Galion | 4.7° N 52.4° W |  |  |
|  | *rosina* | 2097 | Panama | El Renacer, Gamboa | 9° N 79° W | Pacific |  |
|  |  | 1023 | Panama | Camino de las cruces | 9° N 79° W |  |  |
|  |  | 2071 | Panama | El Renacer, Gamboa | 9° N 79° W |  |  |
|  | *cythera* | 8074 | Ecuador | Mindo - Cinto Road | 0.648° S 78.78° W | Pacific |  |
|  |  | 8073 | Ecuador | Mindo - Cinto Road | 0.648° S 78.78° W |  |  |
|  |  | 8237 | Ecuador | Pedro Vicente Maldonado (pichincha) | 0.12° N 79.59° W |  |  |
|  | *vulcanus* | 710 | Colombia | Rio Bravo-Rio Calima (Valle del Cauca) | 3° N 76° W | Pacific |  |
|  |  | 712 | Colombia | Rio Bravo-Rio Calima (Valle del Cauca) | 3° N 76° W |  |  |
|  | *amaryllis* | 1287 | Peru | Tarapoto, Rio Shilcayo | 2° S 74° W | East Andes foothills |  |
|  |  | 1285 | Peru | Tarapoto, Rio Shilcayo | 2° S 74° W |  |  |
|  |  | 1282 | Peru | Tarapoto, Rio Shilcayo | 2° S 74° W |  |  |
|  | *aglaope* | 1635 | Peru | Above Pachiza | 9° S 75°W | Amazonia |  |
|  |  | 1432 | Peru | Yuracyacu trail, Km 62 Tarapoto-Yarimaguas | 7° S 77° W |  |  |
|  |  | 1385 | Peru | Tarapoto Yarimaguas Km 58 | 5.9° S 76°W |  |  |
|  | *maletti* | 16540 | Ecuador | Y de misahualli | 1.06° S 77.6° W | Amazonia |  |
|  |  | 17402 | Ecuador | San Pedro de Arajuno | 1.09° S 77.6° W |  |  |
|  |  | 17390 | Ecuador | San Pedro de Arajuno | 1.09° S 77.6° W |  |  |
|  |  | 1289 | Colombia | Paraiso (Caqueta) | 1° N 75° W |  |  |
|  |  | 1261 | Colombia | Paraiso (Caqueta) | 1° N 75° W |  |  |
|  |  | 2312 | Colombia | Finca C. Piñacué (Caqueta) | 1° N 75° W |  |  |
|  | *plesseni* | 9156 | Ecuador | El Topo, Puyo Road | 1.39° S 78.17° W | East Andes foothills |  |
|  |  | 9194 | Ecuador | El Topo, Puyo Road | 1.39° S 78.17° W |  |  |
|  |  | 9228 | Ecuador | Rio Anso, near Mera | 1° S 78° W |  |  |
|  | *ecuadoriensis* | 9112 | Ecuador | Old Zamora Road | 4º S 78º W | Amazonia |  |
|  |  | 9115 | Ecuador | Old Zamora Road | 4º S 78º W |  |  |
|  |  | 9120 | Ecuador | Old Zamora Road | 4º S 78º W |  |  |
|  | *thelxiopeia* | 13475 | French Guiana | Maripasoula | 3º N 54º W | Guiana shield |  |
|  |  | 13566 | French Guiana | Maripasoula | 3º N 54º W |  |  |
|  | *amandus* | 2221 | Bolivia | Angostura Via Santa Cruz-Samaipata | 18° S 63° W | East Andes foothills |  |
|  |  | 2222 | Bolivia | Angostura Via Santa Cruz-Samaipata | 18° S 63° W |  |  |
|  |  | 2227 | Bolivia | Angostura Via Santa Cruz-Samaipata | 18° S 63° W |  |  |
|  |  | 2228 | Bolivia | Angostura Via Santa Cruz-Samaipata | 18° S 63° W |  |  |
