# Supplementary material for: Adaptive Introgression across Species Boundaries in Heliconius Butterflies
Source: PLoS Genet. 2012 Jun 21;8(6):e1002752. doi: 10.1371/journal.pgen.1002752 (PMC3380824; doi:10.1371/journal.pgen.1002752)
Supplement: Table S2 — Primers and PCR information. (DOC) [file pgen.1002752.s004.doc]

**Table S2. Primers and PCR information**

| **Gene/Fragment** | **Type** | | **Primer Forward (5' to 3')** | **Primer Reverse (5' to 3')** | **Tm** | **Size** |
| --- | --- | --- | --- | --- | --- | --- |
| COI* | Unlinked to red colouration | Mitochondrial | CAACATTTATTTTGATTTTTTGG | TAGGATTAGCTGGAATACC | 48 | 900 bp |
| GAPDH |  | Nuclear coding | AARGCTGGRGCTGAATATGT | GWTTGAATGTACTTGATRAGRTC | 48 | 702 bp |
| Hsp90 |  | Nuclear coding | AAATGCCAGAGGAAAAAATG | GATAAGGTCTTCACAGTTGT | 50 | 1000 bp |
| Kinesin | Linked to red colouration | Nuclear coding | CACTCGATTCGCCTCAAGTTCAAAGAT | GGGAGCCGGTTTATCTAATGTC | 48 | 730 bp |
| Hm01012 |  | Nuclear coding | TCCAACCATGAACATGATAAAA | CTGCAAGCGAATTCACTCAT | 55 | 230 |
| HmB_449k |  | Nuclear non coding | CGGTTCCCGATTGTGATTA | CTAAAATTTGCGTAGCGATCA | 55 | 770 bp |
| HmB_453k |  | Nuclear non coding | TGAAGTAAGCGAGGCCATTC | CTGGTGAAGCGAACAAGACA | 55 | 800 bp |
| Optix |  | Nuclear coding | AATGCGTCCAGAAGGCATAC | CCGAGAGCTCTACTCGATCC | 55 | 800 bp |
| HmB_520k |  | Nuclear non coding | GTGTAAACACGCTCCGTTCC | ATCCGCGAATGAAATGCTTG | 55 | 350 bp |

***** Primers reported by Beltran et al. 2002
