# Supplementary material for: Adaptive Introgression across Species Boundaries in Heliconius Butterflies
Source: PLoS Genet. 2012 Jun 21;8(6):e1002752. doi: 10.1371/journal.pgen.1002752 (PMC3380824; doi:10.1371/journal.pgen.1002752)
Supplement: Table S3 — Population size parameter estimated by IM. (DOC) [file pgen.1002752.s005.doc]

**Table S3. Population size parameters inferred with IM**

| **Phenotype** | **Postman** | | **Rays** | |
| --- | --- | --- | --- | --- |
| **Species** | ***H. melpomene*** | ***H. timareta*** | ***H. melpomene*** | ***H. timareta*** |
| **Population size (q)** | 2111109 | 530541 | 1838026 | 792374 |
| **Lower 90% HPD** | 1914087 | 333066 | 1359550 | 608254 |
| **Upper 90% HPD** | 2822439 | 793379 | 1907492 | 1042251 |
